# Supplementary material for: Fish Consumption and Risk of Stroke in Chinese Adults: A Prospective Cohort Study in Shanghai, China
Source: Nutrients. 2022 Oct 12;14(20):4239. doi: 10.3390/nu14204239 (PMC9612352; doi:10.3390/nu14204239)
Supplement: Supplementary file 1 [file nutrients-14-04239-s001.zip › nutrients-1946728-supplementary.pdf]

Supplemental Table S1. Categories and weighted percentage of covariates in the models

Covariates in models	Weighted percentages (%)
Age (years)	Continuous
Gender	
Male	39.71
Female	60.29
Education	
Primary school or below	35.90
Junior high school	39.05
Senior high school	15.47
High school or above	9.59
Marriage status	
Married	92.64
Unmarried	1.50
Divorced and other	5.86
Retired	
Yes	61.27
No	38.73
Smoking status	
Never	77.37
Former	3.66
Current	18.98
Alcohol drinking	
Never	87.49
Former	1.09
Current	11.41
PA levels (METs-min/week)	
Less than 3089	33.33
3090-5040	33.29
More than 5040	33.37
PSQI score	
Less than 2	19.25
2-4	37.83
4 and above	42.91
Energy intake (kcal/day)	
Less than 1257.16	33.33
1257.16-1681.60	33.33
1681.60 and more	33.33
General obesity	
Normal weight	50.75
Overweight	37.18

Obesity	12.07
Fruit (g/day)	
Less than	81.01
200-350	15.51
More than 350	3.47
Vegetables (g/day)	
Less than 300	57.13
300-500	27.14
More than 500	15.73
Peanuts (g/week)	
Less than 50	60.22
50-70	4.57
More than 70	35.21
Whole grains (g/day)	
Less than 50	89.55
50 and more	10.45
Processed meats	
Not eat	52.48
Eat	47.52
Unprocessed meats (g/day)	
Less than 40	38.27
40-75	32.53
More than 75	29.20
Bean products (g/day)	
Less than 30	68.65
30-50	10.28
More than 50	21.08
Salt (g/day)	
5 and less	69.98
More than 5	30.02
Oil (g/day)	
Less than 25	35.28
25-30	15.23
More than 30	49.49
Hypertension	
Yes	63.20
No	36.80
CHD	
Yes	4.25
No	95.75
Diabetes	
Yes	15.01
No	84.99
CKD	

Yes	14.30
No	85.70
Dyslipidemia	
Yes	31.16
No	68.84
COPD	
Yes	0.51
No	99.49
Chronic bronchitis	
Yes	6.58
No	93.42
Asthma	
Yes	2.08
No	97.92
HUA	
Yes	16.80
No	83.20
HHcy	
Yes	23.03
No	76.97

PA, physical activities; METs, metabolic equivalents; PSQI, Pittsburgh Sleep Quality Index; CHD, coronary heart disease; CKD, chronic kidney disease; HUA, hyperuricemia; HHcy, hyperhomocysteinemia; COPD, chronic obstructive pulmonary disease.

Supplemental Table S2. Hazard ratios (95% CIs) of total stroke, ischemic stroke and hemorrhagic stroke by four categories of fish consumption after excluding deaths within the first two years of follow-up

	Fish consumption (g/week)				P for trend
Total stroke	Less than 300	300-450	450-600	More than 600	
Model1	1.00	0.74 (0.60-0.89)	0.79 (0.63-0.99)	0.92 (0.74-1.14)	0.13
Model2	1.00	0.76 (0.63-0.94)	0.84 (0.66-1.07)	1.03 (0.81-1.30)	0.72
Model3	1.00	0.77 (0.63-0.94)	0.84 (0.66-1.06)	1.02 (0.80-1.29)	0.68
Ischemic stroke					
Model1	1.00	0.67 (0.54-0.83)	0.78 (0.61-0.99)	0.83 (0.65-1.05)	0.02
Model2	1.00	0.69 (0.55-0.86)	0.82 (0.63-1.07)	0.92 (0.71-1.20)	0.25
Model3	1.00	0.70 (0.56-0.87)	0.82 (0.63-1.07)	0.91 (0.70-1.19)	0.22
Hemorrhagic stroke					

Model1	1.00	1.16 (0.71-1.88)	0.60 (0.28-1.27)	1.10 (0.61-1.97)	0.90
Model2	1.00	1.29 (0.78-2.13)	0.69 (0.32-1.51)	1.36 (0.71-2.62)	0.55
Model3	1.00	1.28 (0.78-2.12)	0.69 (0.31-1.51)	1.36 (0.70-2.62)	0.56

Linear trend was calculated by treating median values across categories as a continuous variable in the model. Model 1: adjusted for age, sex, educational levels, marital status and retirement status. Model 2: adjusted further for smoking status, alcohol drinking status, physical activity levels, sleep qualities, obesity status, dietary energy intakes and consumption of fruit, vegetables, peanuts, wholegrains, processed and unprocessed meats, bean products, salt and oil. Model 3: adjusted further for histories of chronic diseases, including hypertension, CHD, diabetes, CKD, dyslipidemia, HUA, HHcy, COPD, chronic bronchitis and asthma.

Supplemental Table S3. Hazard ratios (95% CIs) of total stroke, ischemic stroke and hemorrhagic stroke by four categories of fish consumption after excluding the category of shrimp, crab and shellfish

	Fish consumption (g/week)				P for trend
Total stroke	Less than 150	150-300	300-450	More than 450	
Model1	1.00	0.86 (0.73-1.01)	0.73 (0.60-0.90)	0.86 (0.69-1.08)	0.06
Model2	1.00	0.89 (0.74-1.05)	0.78 (0.63-0.97)	0.97 (0.76-1.24)	0.52
Model3	1.00	0.89 (0.75-1.06)	0.77 (0.62-0.96)	0.96 (0.75-1.22)	0.44
Ischemic stroke					
Model1	1.00	0.81 (0.67-0.97)	0.67 (0.54-0.85)	0.82 (0.64-1.05)	0.03
Model2	1.00	0.83 (0.68-1.00)	0.71 (0.56-0.91)	0.91 (0.70-1.20)	0.27
Model3	1.00	0.83 (0.69-1.00)	0.70 (0.55-0.90)	0.90 (0.89-1.18)	0.22
Hemorrhagic stroke					
Model1	1.00	1.12 (0.72-1.74)	0.86 (0.50-1.49)	0.87 (0.46-1.64)	0.49
Model2	1.00	1.26 (0.80-1.99)	1.05 (0.58-1.88)	1.14 (0.57-2.27)	0.85
Model3	1.00	1.25 (0.79-1.97)	1.02 (0.57-1.84)	1.13 (0.57-2.24)	0.89

Linear trend was calculated by treating median values across categories as a continuous variable in the model. Model 1: adjusted for age, sex, educational levels, marital status and

retirement status. Model 2: adjusted further for smoking status, alcohol drinking status, physical activity levels, sleep qualities, obesity status, dietary energy intakes and consumption of fruit, vegetables, peanuts, wholegrains, processed and unprocessed meats, bean products, salt and oil. Model 3: adjusted further for histories of chronic diseases, including hypertension, CHD, diabetes, CKD, dyslipidemia, HUA, HHcy, COPD, chronic bronchitis and asthma.
